# Supplementary material for: Effect of Environmental Enrichment and Herbal Compounds-Supplemented Diet on Pig Carcass, Meat Quality Traits, and Consumers’ Acceptability and Preference
Source: Animals (Basel). 2018 Jul 16;8(7):118. doi: 10.3390/ani8070118 (PMC6071039; doi:10.3390/ani8070118)
Supplement: Supplementary file 1 [file animals-08-00118-s001.pdf]

Supplementary Material S1

Effect of environmental enrichment and herbal compounds-supplemented diet on pig carcass, meat quality traits, and consumers' acceptability and preference.

Nicolau Casal, Maria Font-i-Furnols, Marina Gispert, Xavier Manteca and Emma Fàbrega

Statistical model for carcass quality variables:

$Y_{ijk} = \mu + H_i + E_j + (H \times E)_{ij} + W_k + e_{ijk}$

Y_{ijk} : observation ijk

μ : mean of the observation ijk

H_i : use or not use of herbal compound

E_j : user or not use of environmental enrichment

$(H \times E)_{ij}$: interaction between H and E

W_k : carcass weight as covariate

e_{ijk} : error of the observation ijk

For live weight, carcass weight and carcass yield variables, W was not included in the model as covariate.

Statistical model for meat quality variables:

$Y_{ijkl} = \mu + H_i + E_j + (H \times E)_{ij} + D_k + e_{ijkl}$

Y_{ijkl} : observation ijkl

μ : mean of the observation ijkl

H_i : use or not use of herbal compound

E_j : user or not use of environmental enrichment

$(H \times E)_{ij}$: interaction between H and E

e_{ijkl} : error of the observation ijkl

Dendogram of the cluster analysis. From it, 3 clusters were selected:

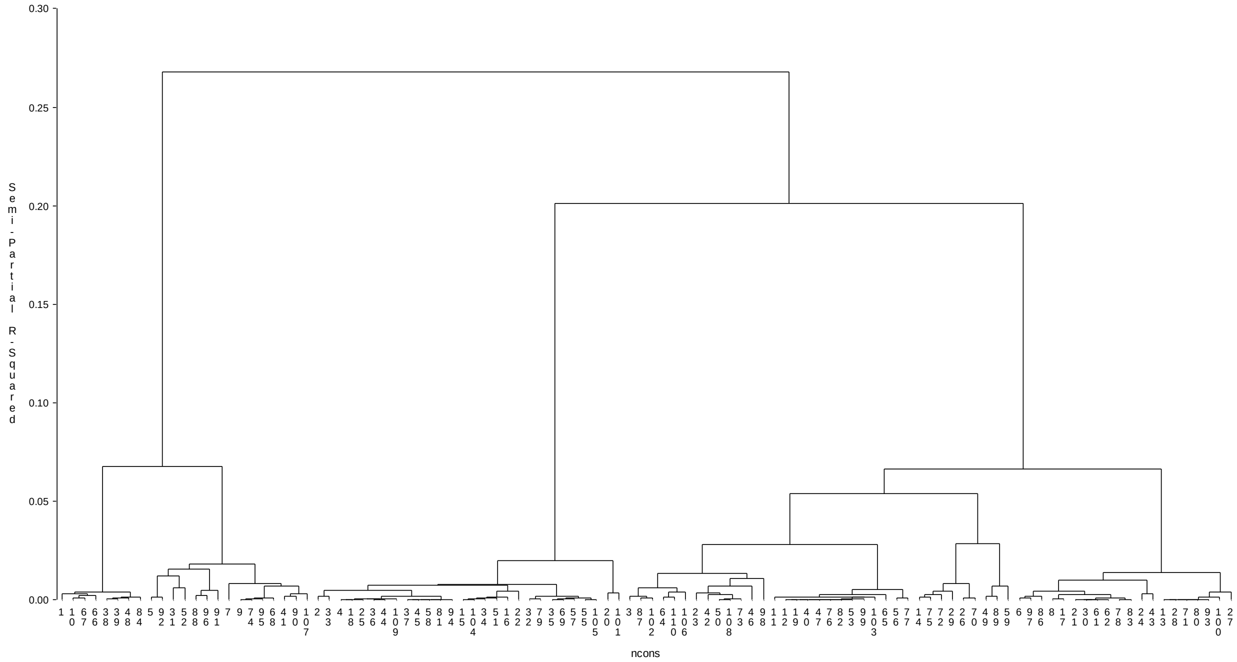

Statistical model for the consumer study analysis:

$Y_{ijkl} = \mu + H_i + E_j + (H \times E)_{ij} + C_k + S_l + e_{ijkl}$

Y_{ijkl}

Y_{ijkl} : observation ijkl

μ : mean of the observation ijkl

H_i : use or not use of herbal compound

E_j : user or not use of environmental enrichment

$(H \times E)_{ij}$: interaction between H and E

C_k : Consumer as random

S_l : Session as blocking effect

e_{ijkl} : error of the observation ijkl
